# Polite Speech Emerges From Competing Social Goals

**DOI:** 10.1162/opmi_a_00035

**Published:** 2020-11

**Authors:** Erica J. Yoon, Michael Henry Tessler, Noah D. Goodman, Michael C. Frank

**Affiliations:** Department of Psychology, Stanford University; 2 Department of Psychology, Stanford University; 3 Department of Brain and Cognitive Sciences, MIT; Department of Psychology, Stanford University; Department of Psychology, Stanford University

**Keywords:** politeness, computational modeling, communicative goals, pragmatics

## Abstract

Language is a remarkably efficient tool for transmitting information. Yet human speakers make statements that are inefficient, imprecise, or even contrary to their own beliefs, all in the service of being polite. What rational machinery underlies polite language use? Here, we show that polite speech emerges from the competition of three communicative goals: to convey information, to be kind, and to present oneself in a good light. We formalize this goal tradeoff using a probabilistic model of utterance production, which predicts human utterance choices in socially sensitive situations with high quantitative accuracy, and we show that our full model is superior to its variants with subsets of the three goals. This utility-theoretic approach to speech acts takes a step toward explaining the richness and subtlety of social language use.

## INTRODUCTION

We don’t always say what’s on our minds. Although “close the window!” could be sufficient, we dawdle, adding “can you please…?” or “would you mind…?” Rather than tell an uncomfortable truth, socially aware speakers exaggerate (“Your dress looks great!”) and prevaricate (“Your poem was so appropriate to the occasion”). Such language use is puzzling for classical views of language as information transfer (Buhler, 1934; Frank & Goodman, 2012; Jakobson, 1960; Shannon, 1948). On the classical view, transfer ought to be efficient and accurate: speakers are expected to choose succinct utterances to convey their beliefs (Grice, 1975; Searle, 1975), and the information conveyed is ideally truthful to the extent of a speaker’s knowledge. Polite speech violates these basic expectations about the nature of communication: it is typically inefficient and underinformative, and sometimes even outright false. Yet even young speakers spontaneously produce requests in polite forms (Axia & Baroni, 1985), and adults use politeness strategies pervasively—even while arguing (Holtgraves, 1997), and even though polite utterances may risk high-stakes misunderstandings (Bonnefon et al., 2011).

If politeness only gets in the way of effective information transfer, why be polite? Most obvious is the fact that we have social relationships to maintain, and most linguistic theories assume speaker behavior is motivated by these concerns, couched as either polite maxims (Leech, 1983), social norms (Ide, 1989), or aspects of a speaker or listener’s identity, known as *face* (Brown & Levinson, 1987; Goffman, 1967). Face-based theories predict that when a speaker’s intended meaning contains a threat to the listener’s face or self-image (and potentially the speaker’s face), her messages will be less direct, less efficient, and possibly untruthful. Indeed, when interpreting utterances in face-threatening situations, listeners readily assume that speakers intend to be polite (Bonnefon et al., 2009). How this socially aware calculation unfolds, however, is not well understood. Adopting an example from Bonnefon et al. (2009), when should a speaker decide to say something false (“Your poem was great!” said of an actually mediocre poem) rather than to tell the truth (“Your poem was bad”) or to be indirect (“Some of the metaphors were tricky to understand”)? How do the speaker’s goals enter into the calculation?

We propose a utility-theoretic solution to the problem of understanding polite language, in which speakers choose their utterances by attempting to maximize utilities that represent competing communicative goals. Under the classical pragmatic view of language production, speakers want to be informative and convey accurate information as efficiently as possible (Goodman & Frank, 2016; Grice, 1975); this desire for informative and efficient communication we call *informational utility*. In addition, speakers may want to be kind and make the listener feel good (i.e., save the listener’s face), for example, by stating positive remarks about the listener. The utility that underlies this goal is a *prosocial utility*.

If a speaker wants to be informative and kind, then she would ideally produce utterances that satisfy both goals. The nuances of reality, however, can make it difficult to satisfy both goals. In particular, when the true state of the world is of low value to the listener (e.g., the listener’s poem was terrible), informational and prosocial goals pull in opposite directions. Informational utility could be maximized by stating the blunt truth (“Your poem was terrible”) but that would very likely hurt the listener’s feelings and threaten the listener’s self-image (low prosocial utility); prosocial utility could be maximized through a white lie (“Your poem was amazing”), but at the cost of being misleading (low informational utility). In such situations, it seems impossible to be both truthful and kind. A first contribution of our work here is to formalize the details of this tradeoff in order to predict experimental data.

A second contribution of our work is to develop and test a new theoretical proposal. We propose that speakers may navigate their way out of the truth-kindness conflict by signaling to the listener that they care about both of the goals, even while they are genuinely unable to fulfill them. We formalize this notion of *self-presentational utility* and show that it leads speakers to prefer indirect speech: utterances that provide less information relative to alternatives with a similar meaning.

We look at indirect speech in this article through negated adjectival phrases (e.g., “It *wasn’t bad*”). The relationship between negation and politeness is a topic of long-standing interest to linguists and psychologists (Bolinger, 1972; Horn, 1989; Stern, 1931; Stoffel, 1901). Comprehending negation, as a logical operation, can be psychologically more complex than comprehending an unnegated assertion, resulting in difficulty in processing of negations (Clark & Chase, 1972; see Nordmeyer & Frank, 2015, for an underlying pragmatic explanation) as well as failure to recognize or recall the asserted content (Lea & Mulligan, 2002; MacDonald & Just, 1989). Our interest in negation, however, is for its information-theoretic properties: negating an assertion that has a specific meaning results in a meaning that is less precise and lower in informativity (e.g., negating “Alex has blue eyes” results in the statement that “Alex has eyes that are some color other than blue”). In our paradigm, we use negation as a way of turning a relatively direct statement (“It was terrible”) into an indirect statement (“It wasn’t terrible”) whose interpretation includes some possibilities that are consistent with or close to the unnegated statement (i.e., the poem was not terrible, but it might still be pretty bad).

Multifactorial, verbal theories—like previous proposals regarding politeness—are very difficult to relate directly to behavioral data. Therefore, to test our hypotheses about the factors underlying the production of polite language (what we refer to as its utility structure), we take a model comparison approach. We do this by formalizing the trade-off between different combinations of speakers’ utilities in a class of probabilistic models of language use (the Rational Speech Act [RSA] framework, Frank & Goodman, 2012; Goodman & Frank, 2016), with a particular focus on models with and without the self-presentational utility. In this framework, speakers are modeled as agents who choose utterances by reasoning about their potential effects on a listener, while listeners infer the meaning of an utterance by reasoning about speakers and what goals could have led them to produce their utterances. These models build on the idea that human social cognition can be approximated via reasoning about others as rational agents who act to maximize their subjective utility (Baker et al., 2009), a hypothesis that has found support in a wide variety of work with both adults and children (e.g., Jara-Ettinger et al., 2016; Liu et al., 2017). Indeed, this class of pragmatic language models has been productively applied to understand a wide variety of complex linguistic behaviors, including vagueness (Lassiter & Goodman, 2017), hyperbole (Kao et al., 2014), and irony (Kao & Goodman, 2015), among others.

## MODEL

**Figure 1.**
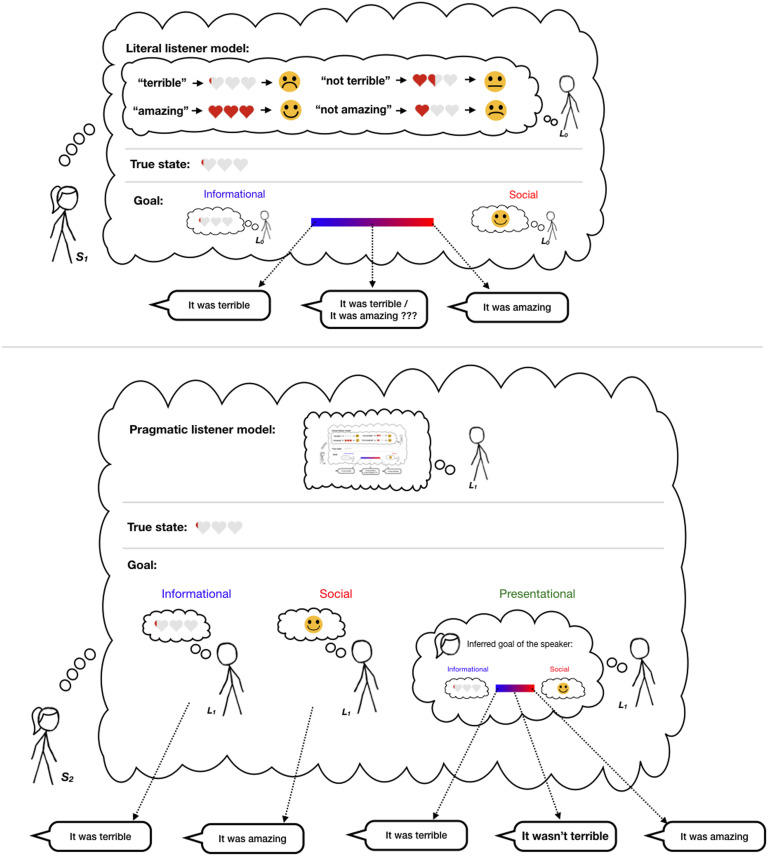
**Diagram of the model, showing *S*_1_ (a first-order polite speaker) and *S*_2_ (a higher-order polite speaker capable of self-presentational goals).** Top: First-order polite speaker (*S*_1_) produces an utterance by thinking about: (1) the true state of the world (i.e., how good a given performance was), (2) the reasoning of a literal listener who updates his beliefs about the true state via the literal meanings of utterances (e.g., “not terrible” means in expectation 1.5 out of 3 hearts) and the effect associated with the state implied by the utterance, and (3) her goal of balancing informational and social utilities. Bottom: Second-order polite speaker (*S*_2_) produces an utterance by thinking about (1) the true state, (2) the pragmatic listener *L*_1_ who updates his beliefs about the true state and the first-order speaker *S*_1_’s goal (via reasoning about the *S*_1_ model), and (3) her goal of balancing informational, prosocial, and self-presentational utilities. Different utterances shown correspond to different weightings of the utility components.

RSA models are defined recursively such that speakers *S* reason about listeners *L*, and vice versa. We use a standard convention in indexing and say a pragmatic listener *L*_1_ reasons about the intended meaning and goals that would have led a speaker *S*_1_ to produce a particular utterance. *S*_1_ reasons about a *literal listener* *L*_0_, who is modeled as attending only to the literal meanings of words (rather than their pragmatic implications), and hence grounds the recursion (Figure 1). The target of our current work is a model of a polite speaker *S*_2_ who reasons about what to say to *L*_1_ by considering some combination of informational, social, and self-presentational goals (Figure 1, bottom).

We evaluate our model’s ability to predict human speaker production behavior in situations where polite language use is expected. Our experimental context involves a speaker (“Ann”) responding to the request of their listener (“Bob”) to evaluate the listener’s (Bob’s) creative product. For instance, Bob recited a poem and asked Ann how good it was. Ann (*S*_2_) produces an utterance *w* based on the true state of the world *s* (i.e., the rating, in her mind, truly deserved by Bob’s poem), a set of goal weights ***ω***, that determines how much Ann prioritizes each of the three possible goals, and a goal weight *ϕ* to project to the listener (details below). Following standard practice in RSA models, Ann’s production decision is softmax, which interpolates between choosing the maximum-utility utterance and probability matching (via speaker optimality parameter *α*; Goodman & Stuhlmüller, 2013):

$$P_{S_{2}}(w|s,\omega )\propto exp[\alpha \cdot U_{total}(w;s;\omega ;\phi )]$$ (1)

We posit that a speaker’s utility contains distinct components that represent three possible goals that speakers may entertain: informational, prosocial, and presentational. These components were determined based on multiple iterations of preliminary experiments, after which we conducted the preregistered test of our specified model with the specific utilities that we report following (Yoon et al., 2016, 2017).

We take the total utility *U*_*total*_ of an utterance to be the weighted combination of the three utilities minus the utterance cost *C*(*w*), which is used to capture the general pressure toward economy in speech (e.g., longer utterances are more costly):

$$U_{total}(w;s;\omega ;\phi )=\omega_{inf}\cdot U_{inf}(w;s)+\omega_{soc}\cdot U_{soc}(w)+\omega_{pres}\cdot U_{pres}(w;\phi )-C(w).$$ (2)

First, a speaker may desire to be epistemically helpful, modeled as standard *informational utility* (*U*_*inf*_). The informational utility indexes the utterance’s negative *surprisal*, or amount of information the listener (*L*_1_) would still not know about the state of the world *s* after hearing the speaker’s utterance *w* (e.g., how likely is Bob to guess Ann’s actual opinion of the poem): *U*_*inf*_(*w*) = *ln*(*P*_*L*_1__(*s*|*w*)).

Speakers who optimize for informational utility produce accurate and informative utterances while those who optimize for social utility produce utterances that make the listener feel good. We define *social utility* (*U*_*soc*_) to be the expected subjective utility of the state *V*(*s*) implied to the pragmatic listener by the utterance: *U*_*soc*_(*w*) = 𝔼_*P*_*L*_1__(*s*|*w*)_ [*V*(*s*)]. The subjective utility function *V*(*s*) is a mapping from states of the world to subjective values, which likely varies by culture and context; we test our model when states are explicit ratings (e.g., numbers on a 4-point scale) and we assume the simplest positive linear relationship between states *s* and values *V*(*s*), where the subjective value is the numerical value of the state (i.e., the number of hearts). For example, Bob would prefer to have written a poem deserving 4 points (visualized as 3 hearts) rather than 1 point (visualized as 0 hearts) and the strength of that preference is 4-to-1.

Listeners who are aware that speakers can be both kind and honest could try to infer the relative contribution of these two goals to the speaker’s behavior (e.g., by asking himself: “was Ann just being nice?”). Thus, we use a pragmatic listener model who has uncertainty about the speaker’s goal weight (relative contribution of niceness vs. informativeness) in addition to their uncertainty about the state of the world (number of hearts; Equation 4). A speaker gains presentational utility when her listener believes she has particular goals, represented by a mixture parameter *ϕ* weighting the goals to be genuinely informative vs. kind.

A sophisticated speaker can then produce utterances in order to appear *as if* she had certain goals in mind, for example, making the listener think that the speaker was being both kind and informative. Such a *self-presentational* goal may be the result of a speaker trying to save their own face (*I want the listener to see that I’m a decent person*) and can result in different speaker behavior depending on the intended, projected goal of the speaker (e.g., *I want the listener to think I’m being honest* vs. *nice* vs. *both*).^1^

The extent to which the speaker *projects* a particular goal to the listener (e.g., to be kind) is the utterance’s *presentational utility* (*U*_*pres*_). Formally,

$$U_{pres}(w;\phi )=ln(P_{L_{1}}(\phi |w))=ln\int_{s}P_{L_{1}}(s,\phi |w).$$ (3)

The speaker projects a particular weighting of informational vs. social goals (*ϕ*) by considering the beliefs of listener *L*_1_, who hears an utterance and jointly infers the speaker’s utilities and the true state of the world:

$$P_{L_{1}}(s,\phi |w)\propto P_{S_{1}}(w|s,\phi )\cdot P(s)\cdot P(\phi ).$$ (4)

The presentational utility *U*_*pres*_ is the highest order term of the model, defined only for a speaker thinking about a listener who evaluates a speaker (i.e., defined for the second-order speaker *S*_2_, but not the first-order speaker *S*_1_). Only the social and informational utilities are defined for the first-order *S*_1_ speaker (via reasoning about *L*_0_); thus, *S*_1_’s utility weightings can be represented by a single number, the mixture parameter *ϕ*. Definitions for *S*_1_ and *L*_0_ otherwise mirror those of *S*_2_ and *L*_1_ and we use the same speaker optimality parameter for *S*_1_ and *S*_2_ for simplicity; these submodels are defined in the next section and appear in more detail in the Supplemental Materials (Yoon et al., 2020). The complete model specification is in Figure 4.

Within our experimental domain, we assume there are four possible states of the world corresponding identically to the value placed on a particular referent (e.g., the 1-to-4 numeric rating of the poem the speaker is commenting on), represented in terms of numbers of hearts (Figure 1): *S* = *s*_0_, …, *s*_3_. In the experiment, participants are told that the listener has no idea about the quality of the product; thus, both listener models *L*_1_ and *L*_0_ assume uniform priors P(s) over the four possible heart states. The pragmatic listener’s prior distribution over the first-order speaker’s utility weights *P*(*ϕ*) encodes baseline assumptions about the relative informativeness vs. niceness listener’s expect, which also plausibly varies by culture and context; for simplicity, we assume this distribution to be uniform over the unit interval (0, 1). The set of utterances for the speaker models *S*_2_ and *S*_1_ is a set of four utterances that intuitively correspond to each unique state as well as their respective negatives {*terrible*, *bad*, *good*, *amazing*, *not terrible*, *not bad*, *not good*, *and not amazing*}; the cost of an utterance is its length in terms of number of words (i.e., utterances with negation are costlier than those without negation) scaled by a free parameter. We implemented this model using the probabilistic programming language WebPPL (Goodman & Stuhlmüller, 2014) and a demo can be found at http://forestdb.org/models/politeness.html.

## MODEL PREDICTIONS

**Figure 2.**
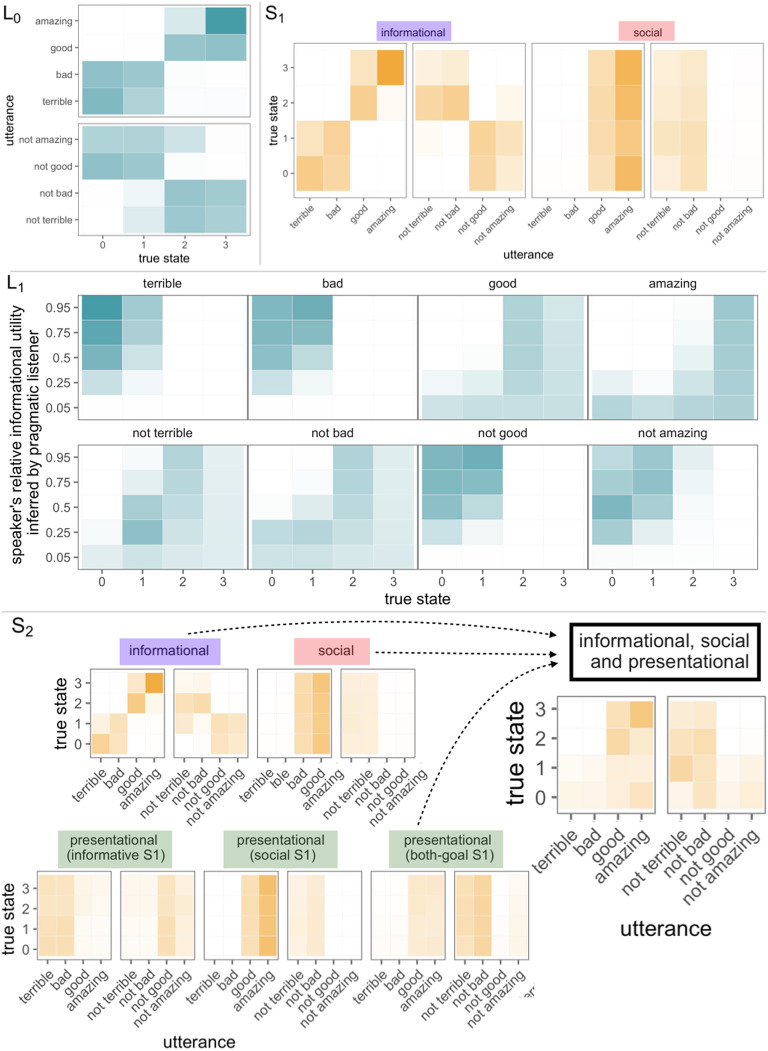
**Model overview with schematic predictions.** Color saturation indicates probability (listener models) or utility (speaker models). Top left (*L*_0_): Literal listener posterior probability distribution over the true state s (*x*-axis) given utterances (*y*-axis). Top right (*S*_1_): the first-order speaker’s utility of utterances w (*x*-axis) for different states s (*y*-axis) given either the informational (*ϕ* = 1) or social goal (*ϕ* = 0; facets). Informational utility tracks the literal meanings and varies by true state; social utility favors utterances that signal higher valued states. Middle (*L*_1_): Politeness-aware listener’s joint posterior distribution over the true state s (*x*-axis) and S_1_ utility weighting *ϕ* (*y*-axis; higher value indicates greater weight on informational utility) given utterances w (facets). Bottom (*S*_2_): Second-order speaker’s utility of utterances (*y*-axis) for different states (*x*-axis) and different goals *ω* (facets). Informational utility tracks the literal meanings and varies by true state; social utility favors utterances that signal high-valued states; three versions of self-presentational utility are shown, corresponding to whether the speaker wants to project informativeness (*ϕ* = 1), kindness (*ϕ* = 0), or a balance (*ϕ* = 0.3). Only the balanced self-presentational speaker shows a preference for indirect speech. The right-most facet shows *S*_2_’s utterance preferences when they want to balance all three utilities (informational, social, and presentational to project informativeness and kindness).

The behavior of the model can be understood through increasing levels of recursive reasoning. To ground the recursion, we have the literal listener model *L*_0_: a simple Bayesian agent who updates their prior beliefs over world states P(s) (assumed to be uniform) with the truth-functional denotation of the utterance *w* according to the lexicon 𝓛: *P*_*L*_0__(*s*|*w*) ∝ 𝓛(*s*) * *P*(*s*) (i.e., the utterance’s literal meaning). Our lexicon 𝓛 assumes soft-semantic meanings, which we elicit empirically in a separate experiment (*N* = 51, see Supplemental Materials, Yoon et al., 2020. For example, the utterance “good” is compatible with both the two- and three-heart states, while “not terrible” is also compatible with states two and three, though also to some extent with the one-heart state (Figure 2, top left).

The first-order speaker *S*_1_ chooses utterances given a utility function with two components defined in terms of the literal listener: informational and social utility. *Informational utility* (*U*_*inf*_) is the amount of information about the world state conveyed to the literal listener *L*_0_ by the utterance *w*; for example, the highest information utterance associated with the two-heart state is “good”; the best way to describe the zero-heart state is “terrible” (Figure 2, top right; left facet). *Social utility* (*U*_*soc*_) is the expected subjective utility of the world state inferred by the literal listener *L*_0_ given the utterance *w*, which does not depend on the true state.^2^ For instance, the highest social utility utterance is “amazing,” because it strongly implies that the listener is in the three-heart state; negated negative utterances like “not bad” also have some degree of social utility, because they imply high heart states, albeit less directly (Figure 2, top right; right facet). The speaker combines these utilities assuming some weighting *ϕ* and subtracts the cost of the utterance (defined in terms of the length of the utterance) in order to arrive at an overall utility of an utterance for a state and a goal-weighting: *U*(*w*; *s*; *ϕ*) = *ϕ* · *ln*(*P*_*L*_0__(*s*|*w*)) + (1 − *ϕ*) · 𝔼_*P*_*L*_0__(*s*|*w*)_ [*V*(*s*)] − *C*(*w*). The speaker then chooses utterances *w* softmax rationally given the state *s* and his goal weight mixture *ϕ*:

$$P_{S_{1}}(w|s,\phi )\propto exp[\alpha \cdot U[s;w;\phi ]].$$ (5)

The pragmatic listener model *L*_1_ reasons jointly about both the true state of the world and the speaker’s goals (Figure 2, middle). Upon hearing “[Your poem was] amazing,” the listener faces a tough credit-assignment problem: The poem could indeed be worthy of three hearts, but it is also possible that the speaker had strong social goals and then no inference about the quality of the poem is warranted. Hearing “[Your poem] was terrible,” the inference is much easier: the poem is probably truly terrible (i.e., worthy of zero hearts) and the speaker probably does not have social goals. Negation makes the interpreted meanings less precise and hence, inferences about goals are also fuzzier: “not amazing” can be seen as a way of saying that the poem was worthy of zero hearts or one heart, which satisfies some amount of both social and informational goals. “Not bad” is less clear: the speaker could be being nice and the poem was actually worthy of zero or one hearts (i.e., it was bad) or the speaker could be being honest (i.e., it was not bad) and the poem was worth two hearts.

The second-order pragmatic speaker model (*S*_2_) reasons about the pragmatic listener *L*_1_ to decide which utterances to produce based on both the true state of the world and the speaker’s goals (Figure 2, bottom). The informational and social utilities of the second-order speaker mirror those of the first-order speaker: Direct utterances are more informative than those involving negation, and utterances that signal many hearts are more prosocial.^3^ The interesting novel behavior of this level of recursion comes from the different flavors of the self-presentational goal (Figure 2, bottom). When the second-order pragmatic speaker wants to *project* kindness (i.e., appear prosocial) they even more strongly display the preference for utterances that signal positive states (i.e., they are over-the-top positive). When the speaker wants to project honesty and informativeness, they take the exact opposite strategy, producing utterances that cannot be explained by virtue of social utility: direct, negative utterances (e.g., “it was terrible”). Finally, the speaker may present themselves in more subtle ways (e.g., intending to convey they are both kind and honest): This goal uniquely leads to the indirect, negative utterances (e.g., “not terrible,” “not bad”) having high utility. These utterances are literally incompatible with low-heart states, but are also not highly informative; this unique combination is what gives rise to the subtle inference of a speaker who cares about both goals.

## EXPERIMENT: SPEAKER PRODUCTION TASK

We conducted a direct test of our speaker production model and its performance in comparison to a range of alternative models, by instantiating our running example in an online experiment. We developed the preceding model iteratively on the basis of a sequence of similar experiments, but importantly, the current test was fully preregistered and confirmatory. All data analytic models and our full model comparison approach were registered ahead of time to remove any opportunities for overfitting the behavioral data through changes to the model or the evaluation.

### Participants

The 202 participants were those with IP addresses in the United States and were recruited on Amazon’s Mechanical Turk.

### Design and Methods

Participants read scenarios with information on the speaker’s feelings toward some performance or product (e.g., a poem recital), on a scale from zero to three hearts (e.g., one out of three hearts; *true state*). For example, one trial read: *Imagine that Bob gave a poem recital, but he didn’t know how good it was. Bob approached Ann, who knows a lot about poems, and asked* “How was my poem?” Additionally, we manipulated the speaker’s goals across trials: to be *informative* (“give accurate and informative feedback”); to be *kind* (“make the listener feel good”); or to be *both* informative and kind simultaneously. Notably, we did not mention a self-presentational goal to participants; rather, we hypothesize this goal would arise spontaneously from a speaker’s inability to achieve the first-order goals of niceness and honesty (i.e., if a speaker wants to, but can’t, be both honest and nice, they would instead try to signal that they care about both goals). We hypothesized that each of the three experimentally induced goals (*informative*, *kind*, *both*) would induce a different tradeoff between the informational, prosocial, and self-presentational utilities in our model.

**Figure 3.**
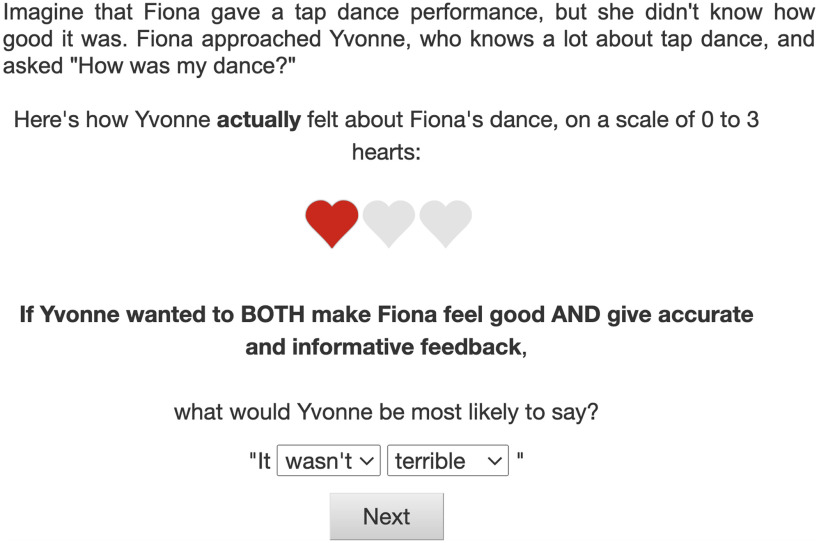
**Example of a trial in the speaker production task.** Trial shows a true state of 1 heart and in the both-goal condition.

Each participant read 12 scenarios, depicting every possible combination of the three goals and four states. The order of context items was randomized, and there were a maximum of two repetitions of each context item per participant. In a single trial, each scenario was followed by a question that read, “If Ann wanted to make Bob feel good but not necessarily give informative feedback (or to give accurate and informative feedback but not necessarily make Bob feel good, or BOTH make Bob feel good AND give accurate and informative feedback), what would Ann be most likely to say?” Participants indicated their answer by choosing one of the options on the two dropdown menus, side-by-side, one for choosing between *It was* vs. *It wasn’t* and the other for choosing among *terrible*, *bad*, *good*, and *amazing* (Figure 3).

### Behavioral Results

Our primary behavioral hypothesis was that speakers describing bad states (e.g., a poem deserving zero hearts) with goals to be both informative and kind would produce more indirect, negative utterances (e.g., It wasn’t terrible). Such indirect speech acts both save the listener’s face and provide some information about the true state, and thus, are what a socially conscious speaker would say (Figure 2, bottom). This prediction was confirmed, as a Bayesian mixed-effects model predicts more negation as a function of true state and goal via an interaction: A speaker with both goals to be informative and kind produced more negation in worse states compared to a speaker with only the goal to be informative (posterior mean *M* = −1.33, with 95% Bayesian credible interval of [−1.69, −0.98]) and goal to be kind (*M* = −0.50, [−0.92, −0.07]). Rather than eschewing one of their goals to increase utility along a single dimension, participants chose utterances that jointly satisfied their conflicting goals by producing indirect speech.

### Model Results

**Figure 4.**
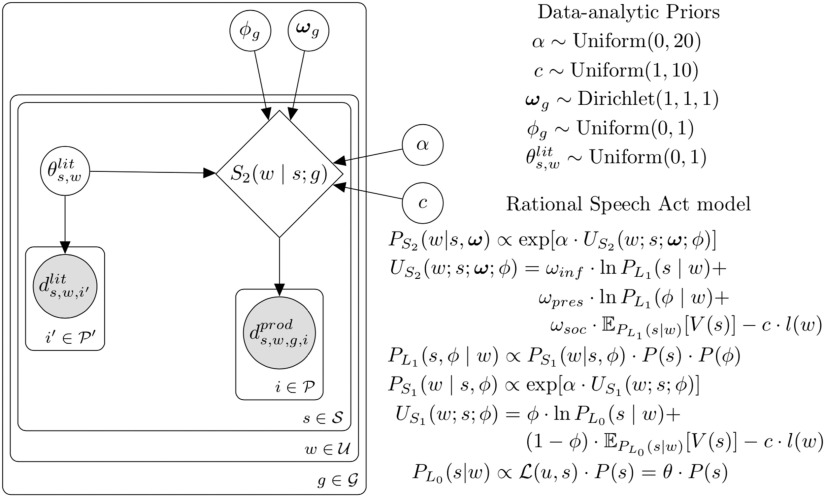
**Graphical model representing our Bayesian data analytic approach for the full three-component model (other models contain subsets of the parameters shown).** *S*_2_ represents the RSA speaker model defined by Equation 1, which is used to predict the production responses *d*^*prod*^ of each participant *i*, for each state *s* (number of hearts), for each utterance *w*, in each goal condition *g*. The RSA speaker model takes as input the literal meaning variables *θ*, which additionally are used to predict the literal meaning judgments *d*^*lit*^ assuming a Bernoulli linking function. Additionally, the RSA model takes the speaker’s goal weights *ω* and intended presentational goal weight *ϕ*, which are inferred separately for each goal condition *g*. Finally, the RSA model uses two global free parameters: the cost of negation *c* (or, utterance length *l* in terms of number of words) and the speaker’s optimality parameter *α*. Minimally assumptive priors over parameters are shown top-right.

**Figure 5.**
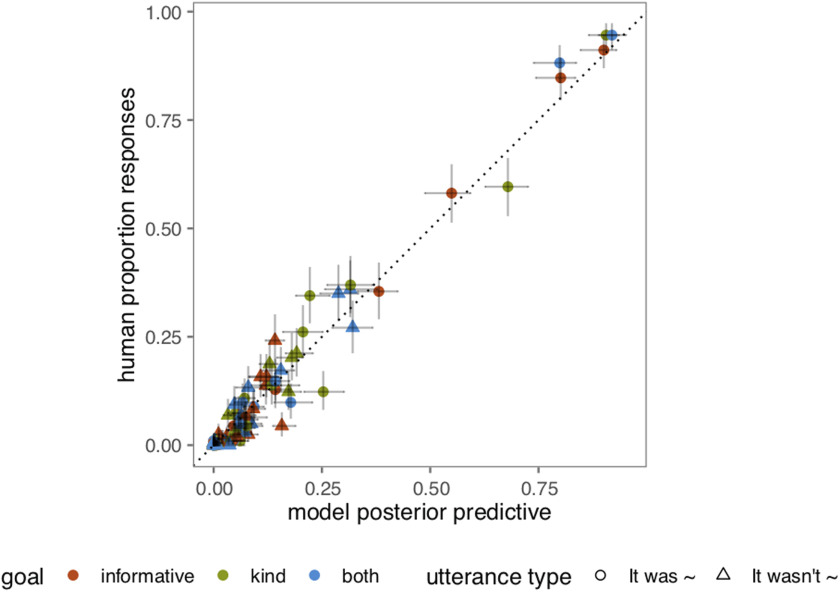
**Full distribution of human responses vs. model predictions.** Error bars represent 95% confidence intervals for the data (vertical) and 95% highest density intervals for the model (horizontal).

We assume our experimental goal conditions (informative vs. kind vs. both) induce a set of weights over the utilities ***ω*** in participants’ utterance production model. In addition, the self-presentational utility is defined via a communicated social weight *ϕ* (i.e., the mixture of informative vs. social that the speaker is trying to project). The mapping from social situations into utility weights and communicated social weight is a complex mapping, which we do not attempt to model here; instead, we infer these parameters for each goal condition from the data. We additionally infer the literal meanings (i.e., the semantics) of the words as interpreted by the literal listener *L*_0_ with the additional constraint of literal meaning judgments from an independent group of participants (see Supplemental Materials: Literal Semantic Task section, Yoon et al., 2020). Finally, the RSA model has two global free parameters: the softmax speaker optimality *α* and utterance cost of negation *c*, which we infer from the data (Figure 4). We implement this data analytic model for each of the alternative models and infer the parameters using Bayesian statistical inference (Lee & Wagenmakers, 2014). We use uninformative priors over ranges consistent with the prior literature on RSA models: $\theta_{s,w}^{lit}$ ∼ Uniform(0, 1), *ϕ*_*g*_ ∼ Uniform(0, 1), ***ω***_*g*_ ∼ Dirichlet(1, 1, 1), *a* ∼ Uniform(0, 20), *c* ∼ Uniform(1, 10). This analysis tells us which, if any, of these models can accomodate all of the patterns in the empirical data. The posterior predictions from the three-utility polite speaker model (informational, social, presentational) showed a very strong fit to participants’ actual utterance choices, *r*^2^(96) = 0.97; Figure 5. Other models (e.g., informational + presentational), however, show comparably high correlations to the full data set; correlations can be inflated through the presence of many zeros (or ones) in the data set, which our data contains since certain utterance choices are almost never selected in state-goal combinations. Thus, we compare model variants using a bonafide model comparison technique, Bayes Factors, which balance predictive accuracy with model complexity in quantifying the goodness of fit of a model.

**Figure 6.**
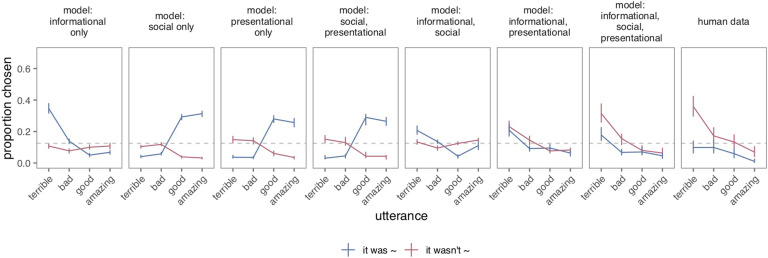
**Predictions for proportion of utterances chosen by pragmatic speaker from possible model variants (left) and human data (rightmost), given true state of 0 hearts (on a scale of 0 to 3) and speaker with both goals to be informative and kind.** Gray dotted line indicates chance level at 12.5%. Only the full model (informational, social, presentational) exhibits a strong preference for negated negative utterances (not terrible, not bad) that is a signature of the human data.

Bayes factors compare the likelihood of the data under each model, averaging over the prior distribution of the model parameters; by averaging over the prior distribution over parameters, Bayes factors penalize models with extra flexibility because increasing the flexibility of the model to fit more data sets decreases the average fit of the model to a particular data set (Lee & Wagenmakers, 2014), capturing the intuition that a theory that can predict anything predicts nothing. That is, simply because a model has more parameters and can explain more of the variance in the data set does not entail that it will assign the highest marginal likelihood to the actual data. Here, however, both the variance explained and marginal likelihood of the observed data were the highest for the full model: The full model was at least 5 ×10^4^ times better at explaining the data than the next best model (Table 1). Only the full model captured participants’ preference for negation when the speaker wanted to be informative and kind about truly bad states, as hypothesized (Figure 6). In sum, the full set of informational, social, and presentational utilities were required to fully explain participants’ utterance choices.

**Table 1.** Comparison of variance explained for each model variant and log Bayes factors quantifying evidence in favor of alternative model in comparison to the full model (informational, social, presentational).

**Model**	**Variance explained**	**Log BF**
informational, social, presentational	0.97	–
informational, presentational	0.96	−11.14
informational, social	0.92	−25.06
social, presentational	0.23	−864
presentational only	0.23	−873.83
social only	0.22	−885.52
informational only	0.83	−274.89

The utility weights inferred for the three-utility model (Table 2) provide additional insight into how polite language use operates in our experimental context and possibly beyond. As expected, the weight on social utility (*ω*_*soc*_) is highest when the speaker is trying to *be kind* and lowest when the speaker is *being informative*. Informational utility (*ω*_*inf*_) is highest when the goal is to be informative or *informative and kind* (“both goal”). The weight on projecting kindness (*ω*_*pres*_) is also highest for the *informative* and the *both-goal* conditions, though the degree of kindness being projected (*ϕ*) varies between these conditions: A greater degree of kindness is projected in the *both-goal* relative to the *informative* condition. In all conditions, however, the presentational utility has a high weight, suggesting that managing the listener’s inferences about oneself was integral to participants’ decisions in the context of our communicative task. Overall then, our condition manipulation altered the balance between these weights, but all utilities played a role in all conditions.

**Table 2.** Maximum a posteriori (MAP) estimates for inferred goal weight (*ω*_*g*_) and speaker-projected informativity-niceness weight (*ϕ*) parameters from all model variants with more than one utility.

**Model (utilities)**	**Goal**	***ω***_***inf***_	***ω***_***soc***_	***ω***_***pres***_	*ϕ*
informational, social, presentational	both	0.36	0.11	0.54	0.36
informational, social, presentational	informative	0.36	0.02	0.62	0.49
informational, social, presentational	social	0.25	0.31	0.44	0.37
informational, presentational	both	0.64	–	0.36	0.17
informational, presentational	informative	0.77	–	0.23	0.33
informational, presentational	social	0.66	–	0.34	0.04
informational, social	both	0.54	0.46	–	–
informational, social	informative	0.82	0.18	–	–
informational, social	social	0.39	0.61	–	–
social, presentational	both	–	0.38	0.62	0.55
social, presentational	informative	–	0.35	0.65	0.75
social, presentational	social	–	0.48	0.52	0.66

## DISCUSSION

Politeness is puzzling from an information-theoretic perspective. Incorporating social motivations into theories of language use adds a level of explanation, but so far such intuitions and observations have resisted both formalization and precise testing. We presented a set of utility-theoretic models of language use that captured different proposals about the interplay between competing informational, social, and presentational goals. Our full model instantiated a novel theoretical proposal, namely, that indirect speech is a response to the conflict between informational and social utilities that preserves speakers’ self-presentation. Our confirmatory test of the comparison between these models then provided experimental evidence that the full model best fit participants’ judgments, even accounting for differences in model complexity.

The most substantial innovation in our full model is the formalization of a self-presentational utility, defined only for a speaker who reasons about a listener who reasons about a speaker. We hypothesized that a speaker who prioritizes presentational utility will tend to produce more indirect speech (negation in our experimental paradigm). Indeed, this is consistent with previous work showing that people prefer to use negation (“that’s not true” as opposed to “that’s false”) when prompted to speak more “politely” (Giora et al., 2005) and that utterances involving negation tend to be interpreted in a more mitigated and hedged manner compared to direct utterances (Colston, 1999). It also may help explain the phenomenon of negative strengthening, where negation of a positive adjective can be interpreted in a rather negative manner (e.g., “He’s not brilliant” meaning “he is rather unintelligent”; Gotzner et al., 2018; Horn, 1989. Our work builds on this previous work that shows a preference for negation by elucidating the goal-directed underpinnings of this behavior and possible contextual modulation of this preference. An interesting open question is whether other negation-related politeness phenomena (e.g., indirect questions such as “You couldn’t possibly tell me the time, could you?”; Brown & Levinson, 1987) can be derived from the basic information-theoretic goals we formalize.

In order to conduct quantitative model comparisons, we had to abstract away from the richness of natural interactions to create an experiment with repeated trials and a restricted set of utterance choices. Thus, we had to abstract away from the richness of natural interactions. These choices decrease the validity of our experiment. Despite these abstractions, we showed that behavior in the experiment reflected social and informational pressures described in previous theories of polite language, providing some face validity to the responses we collected. With a formal model in hand, it now will be possible to consider relaxing some of the experimental simplifications we put into place in future work. Most importantly, human speakers have access to a potentially infinite set of utterances to select from in order to manage the politeness-related tradeoffs (e.g., *It’s hard to write a good poem, That metaphor in the second stanza was so relatable!*). Each utterance will have strengths and weaknesses relative to the speaker’s goals. Computation in an unbounded model presents technical challenges (perhaps paralleling the difficulty human speakers feel in finding the right thing to say in a difficult situation), and addressing these challenges is an important future direction (see Goodman & Frank, 2016).

For a socially conscious speaker, managing listeners’ inferences is a fundamental task. Our work extends previous models of language beyond standard informational utilities to address social and self-presentational concerns. Further, our model builds upon the theory of politeness as face management (Brown & Levinson, 1987) and takes a step toward understanding the complex set of social concerns involved in face management. This latter point illustrates a general feature of why explicit computational models provide value: only by formalizing the factors in Brown and Levinson’s (1987) theory were we able to recognize that they were an insufficient description of the data we were collecting in previous versions of the current experiment. Those failures allowed us to explore models with a broader range of utilities, such as the one reported here.

Previous game-theoretic analyses of politeness have either required some social cost to an utterance (e.g., by reducing one’s social status or incurring social debt to one’s conversational partner; Van Rooy, 2003) or a separately motivated notion of plausible deniability (Pinker et al., 2008). The kind of utterance cost for the first type of account would necessarily involve higher order reasoning about other agents, and may be able to be defined in terms of the more basic social and self-presentational goals we formalize here. A separate notion of plausible deniability may not be needed to explain most politeness behavior, either. Maintaining plausible deniability is in one’s own self-interest (e.g., due to controversial viewpoints or covert deception) and goes against the interests of the addressee; some amount of utility dis-alignment is presumed by these accounts. Politeness behavior appears present even in the absence of obvious conflict, however: in fact, you might be even more motivated to be polite to someone whose utilities are more aligned with yours (e.g., a friend). In our work here, we show that such behaviors can in fact arise from purely cooperative goals (Brown & Levinson, 1987), though in cases of genuine conflict, plausible deniability likely plays a more central role in communication. Our computational model is also closely related to recent developments in modeling *social meaning* in sociolinguistics, where a speaker chooses how they say something (e.g., “I’m grilling” vs. “I’m grillin”’) in order to convey something about themselves (e.g., social class) to the listener (Burnett, 2019). Unlike a social meaning game, which treats properties of a speaker as first-class targets of communication, our model considers the properties of the speaker as variables that modify the speaker’s utility function, about which the listener can then reason (but see also: Henderson & McCready, 2019; Qing & Cohn-Gordon, 2019).

Utility weights and value functions in our model could provide a framework for a quantitative understanding of systematic cross-cultural differences in what counts as polite. Cultures may place value on satisfying different communicative goals, and speakers in these cultures may pursue those goals more strongly than speakers from other cultures. For example, we found in our model that a speaker who wants to appear informative should speak more negatively than a truly informative speaker; one could imagine runaway effects where a group becomes overly critical from individuals’ desires to appear informative. Culture could also affect the value function *V* that maps states of the world onto subjective values for the listener. For example, the mapping from states to utilities may be nonlinear and involve reasoning about the future; a social utility that takes into account reasoning about the future could help explain why it can often be nice to be informative. Our formal modeling approach, with systematic behavior measurements, provides an avenue toward understanding the vast range of politeness practices found across languages and contexts (Katz, 2005).

Politeness is only one of the ways language use deviates from purely informational transmission. We flirt, insult, boast, and empathize by balancing informative transmissions with goals to affect others’ feelings or present particular views of ourselves. Our work shows how social and self-presentational motives can be integrated with informational concerns more generally, opening up the possibility for a broader theory of social language. A formal account of politeness may also move us closer to courteous computation—to machines that can talk with tact.

## FUNDING INFORMATION

MHT, National Science Foundation (http://dx.doi.org/10.13039/100000001), Award ID: DGE-114747. EJY, Natural Sciences and Engineering Research Council of Canada (http://dx.doi.org/10.13039/501100000038), Award ID: PGSD3-454094-2014. NDG, Office of Naval Research (http://dx.doi.org/10.13039/100000006), Award ID: N00014-13-1-0788. MCF, National Science Foundation (http://dx.doi.org/10.13039/100000001), Award ID: BCS 1456077. NDG, Alfred P. Sloan Foundation (http://dx.doi.org/10.13039/100000879). MHT, National Science Foundation (http://dx.doi.org/10.13039/501100008982), Award ID: 1911790.

## AUTHOR CONTRIBUTIONS

EJY: Conceptualization: Equal; Formal analysis: Equal; Methodology: Lead; Visualization: Equal; Writing - Original Draft: Equal; Writing - Review & Editing: Equal. MHT: Conceptualization: Equal; Formal analysis: Equal; Visualization: Equal; Writing - Original Draft: Equal; Writing - Review & Editing: Equal. NDG: Conceptualization: Supporting; Methodology: Supporting; Supervision: Supporting; Writing - Review & Editing: Supporting. MCF: Conceptualization: Supporting; Formal analysis: Supporting; Methodology: Supporting; Supervision: Lead; Visualization: Supporting; Writing - Original Draft: Supporting; Writing - Review & Editing: Supporting.

## Notes

^1^ In principle, one could continue the recursion hierarchy and define a listener *L*_2_ who reasons about this clever speaker and tries to uncover the goals that the speaker was trying to convey to them; we think such reasoning is reserved for very special relationships and is unlikely to manifest in the more basic acts of polite language use that we study here.^2^ The independence between true state and social utility stems from the assumption of no shared beliefs between speaker and listener about the true state (i.e., the speaker knows the true state and the listener’s priors are independent of the true state). This independence is a deliberate feature of our experimental setup, designed to best disambiguate the models proposed. In future work, it would be important to examine how shared beliefs about the true state may influence the speaker’s utterance choice.^3^ The second-order speaker’s informational utilities take into account the listener’s pragmatic inferences about the speaker’s goals. This reasoning only really affects the utility of “not terrible,” which has higher information for the one-heart state because the pragmatic listener strongly infers that the utterance was produced for social reasons. That is, for the second-order speaker, the utterance “not terrible” is loaded in a way that other utterances are not. An alternative formulation could be proposed by having *S*_2_’s informational utility derived from a pragmatic listener who doesn’t reason about the speaker’s goals (i.e., it compares a posterior on states assuming the speaker was being informative, while independently reasoning about whether the speaker was being informative). An examination of this model is beyond the scope of this article.
