# Supplementary material for: Polite Speech Emerges From Competing Social Goals
Source: Open Mind (Camb). 2020 Nov 1;4:71–87. doi: 10.1162/opmi_a_00035 (PMC7672308; doi:10.1162/opmi_a_00035)
Supplement: Supplementary file 1 [file opmi-04-71-s001.pdf]

Supplementary Materials

Model details

The full, three-component utility model is given by the following set of equations.

$$
\begin{aligned}
P_{L_0}(s|w) &\propto \mathcal{L}(u,s) \cdot P(s) \\
P_{S_1}(w \mid s, \phi) &\propto \exp[\alpha \cdot (\phi \cdot \ln P_{L_0}(s \mid w) + (1-\phi) \cdot \mathbb{E}_{P_{L_0}(s|w)}[V(s)] - C(w))] \\
P_{L_1}(s, \phi|w) &\propto P_{S_1}(w|s,\phi) \cdot P(s) \cdot P(\phi) \\
P_{S_2}(w|s, \boldsymbol{\omega}) &\propto \exp[\alpha \cdot (\omega_{inf} \cdot \ln P_{L_1}(s \mid w) + \omega_{soc} \cdot \mathbb{E}_{P_{L_1}(s|w)}[V(s)] + \omega_{pres} \cdot P_{L_1}(\phi \mid w) - C(w))]
\end{aligned}
$$

The *literal listener* L_0 (Eq.) is a simple Bayesian agent that takes the utterance w to be true to update a prior distribution over world states $P(s)$ and return a posterior distribution over states $P_{L_0}(s|w)$. We assume the prior over world states is uninformative and thus Eq. reduces to $P_{L_0}(s|w) \propto \mathcal{L}(u,s)$. $\mathcal{L}(u,s) = \theta \in [0,1]$ denotes a continuously-valued lexicon where θ is the probability that the utterance u is true of state s; this kind of lexicon is a generalization of the more traditional, binary truth functional semantics (see Degen, Hawkins, Graf, Kreiss, and Goodman (2020) for a discussion of this kind of "soft semantics"). θ is estimated empirically from the Literal Semantics task described in the next section, where it is roughly the proportion of participants who endorse the utterance u in state s in the Literal Semantics task.

The first-order *speaker* S_1 (Eq.) chooses utterances approximately optimally given a utility function, which can be decomposed into two components: informational and social utility. First, informational utility (U_{inf}) is the amount of information a literal listener L_0 would still not know about world state s after hearing a speaker's utterance w, and is given by the log probability of the world state given the utterance $\ln P(s \mid w)$. Second, social utility (U_{soc}) is the

expected subjective utility of the state inferred given the utterance w: $\mathbb{E}_{P_{L_0}(s|w)}[V(s)]$, where $V(s)$ denotes the subjective utility function that maps states of the world s onto subjective values. For this paper, we assume that V is the identity function that returns the number of hearts which defines a state. The overall utility of an utterance subtracts the cost $c(w)$ from the weighted combination of the social and informational utilities, and the speaker then chooses utterances w softmax-optimally given the state s and his goal weight mixture ϕ.

Equations and are described in the main text. Eq. is a model of a pragmatic listener who jointly reasons about the state of the world and the first-order speaker's utility weighting (social vs. informational utility). Again, we assume an uninformative prior over states as well as an uninformed prior over the speakear's utility weights. Eq. is a model of a second-order pragmatic speaker who produces utterances approximately optimally given a three-component utility function: informational, social, and presentational utilities. These utilities are defined with respect to the pragmatic listener L_1 and are computed by marginalizing L_1's joint distribution over states and utility-weights: $P_{L_1}(s|w) = \int_\phi P_{L_1}(s, \phi|w) d\phi$ and $P_{L_1}(\phi|w) = \int_s P_{L_1}(s, \phi|w) ds$. The definitions of social and informational utility mirror those of the first-order speaker, only they are defined with respect to the L_1 distribution as opposed to the L_0 distribution. The self-presentational utility is the negative surprisal of the goal-weight parameter that L_1 reasons about: $\ln P_{L_1}(\phi|w)$. These three utilities are then weighed by a set of three mixture components $\boldsymbol{\omega}$, which are inferred from the data separately for each experimental condition (see main text for details).

Literal semantics task

We probed judgments of literal meanings of the target words assumed by our model and used in our main experiment.

Participants 51 participants with IP addresses in the United States were recruited on Amazon's Mechanical Turk.

Design and Methods We used thirteen different context items in which a speaker evaluated a performance of some kind. For example, in one of the contexts, Ann saw a presentation, and Ann's feelings toward the presentation (true state) were shown on a scale from zero to three hearts (e.g., two out of three hearts filled in red color; see Figure ?? for an example of the heart scale). The question of interest was "Do you think Ann thought the presentation was / wasn't X?" and participants responded by choosing either "no" or "yes." The target could be one of four possible words: *terrible*, *bad*, *good*, and *amazing*, giving rise to eight different possible utterances (with negation or no negation). Each participant read 32 scenarios, depicting every possible combination of states and utterances. The order of context items was randomized, and there were a maximum of four repeats of each context item per participant.

Behavioral results We analyzed the data by collapsing across context items. For each utterance-state pair, we computed the posterior distribution over the semantic weight (i.e., how consistent X utterance is with Y state) assuming a uniform prior over the weight (i.e., a standard Beta-Binomial model). Meanings of the words as judged by participants were as one would expect (Figure 3). Importantly, the task does not elicit alternative-based pragmatic reasoning that would result in pragmatically-enriched meanings (e.g., "good" is interpreted to mean "not amazing"; instead "good" is judged equally true at 2 and 3 heart states)

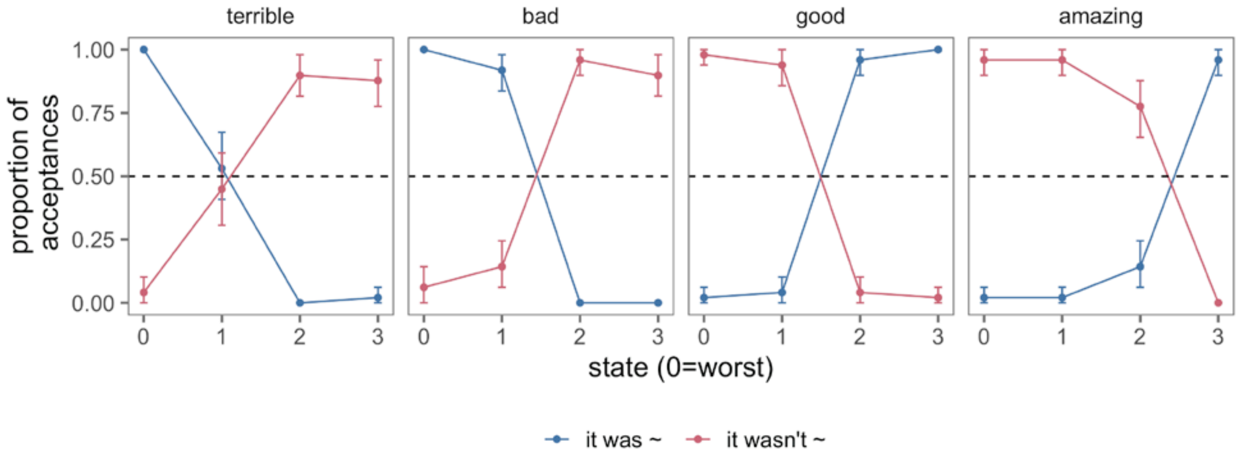

Figure 3 Semantic measurement results. Proportion of acceptances of utterance types (shown in different colors) combined with target words (shown in different facets) given the true state represented on a scale of hearts. Error bars represent 95% confidence intervals.

Full statistics on human data

Table 3

Predictor mean estimates with standard deviation and 95% credible interval information for a Bayesian linear mixed-effects model predicting negation production based on true state and speaker goal (with both-goal as the reference level).

Predictor	Mean	SD	95% CI-Lower	95% CI-Upper
Intercept	0.88	0.13	0.63	1.12
True state	2.18	0.17	1.86	2.53
Goal: Informative	0.47	0.17	0.14	0.80
Goal: Kind	0.97	0.25	0.51	1.49
True state * Informative	-1.33	0.18	-1.69	-0.98
True state * Kind	-0.50	0.22	-0.92	-0.07

We used Bayesian linear mixed-effects models (`brms` package in R; Bürkner, 2017) using crossed random effects of true state and goal with maximal random effects structure (Barr, Levy, Scheepers, & Tily, 2013; Gelman & Hill, 2006). The full statistics are shown in Table 3.

Model fitting and inferred parameters

Table 4

Inferred negation cost and speaker optimality parameters for all model variants.

Model	Cost of negation	Speaker optimality
ninformational only	1.58	8.58
ninformational, presentational	1.89	2.93
ninformational, social	1.11	3.07
ninformational, social, presentational	2.64	4.47
presentational only	2.58	9.58
social only	1.73	7.23
social, presentational	2.49	5.29

Other than speaker goal mixture weights explained in the main text (shown in Table 1), the full model has two global parameters: the speakers' (both S_1 and S_2) soft-max parameter α, which we assume to be the same value, and the utterance cost parameter c. We operationalize utterance cost as a penalty on the number of words in an utterance; since utterances are only one or two words long, and two word-long utterances are those involving the negation particle "not", this cost parameter can also be thought of as a cost of producing a negation particle. We use minimally assumptive priors that are consistent with those used for similar models in this model class: $\alpha \sim \text{Uniform}(0,20)$, $c \sim \text{Uniform}(1,10)$. Cost c is assumed to be greater than 1; values

less than 1 would imply that a two word-long utterance is cheaper than a one-word long utterance (or, that there is a cost to not producing negation). Finally, we incorporate the literal semantics data into the RSA model by maintaining uncertainty about the semantic weight θ of utterance w for state s, for each of the states and utterances, and assuming a Beta-Binomial linking function between these weights and the literal semantics data (see *Literal Semantics task* above). We infer the posterior distribution over all of the model parameters and generate model predictions based on this posterior distribution using Bayesian data analysis (M. D. Lee & Wagenmakers, 2014). We ran 4 MCMC chains for 80,000 iterations, discarding the first 40,000 for burnin. The inferred Maximum A-Posteriori values of parameters are shown in Table 4.

We observe that the posterior distributions of the inferred parameters governing the speaker goal mixture weights are unstable across different MCMC chains. To confirm these observations, we ran 3 additional MCMC chains for 350,000 iterations. The resulting posterior distributions for the utility-weight parameters as a function of the goal condition (a "goal-centric" view) are shown in Figure 4. As can be seen by comparing across the rows of Figure 4, both the values and the relative orderings of the utility-weight parameters vary as a function of the chain. For instance, in one run of the model, the informative goal condition has as its strongest utility weight the presentational utility (chain 1); in another, the strongest utility weight is the informational utility (chain 2); in yet another, the informational and presentational weights are approximately equal in strength (chain 3). At the same time, for the informative goal condition, the social utility weight is always close to 0, in all runs of the model. Indeed, the social utility weight appears most consistent across goal conditions and chains. Further, when we examine the utility weights as a function of goal conditions (a "utility-centric" view), we find other signatures of consistency (Figure 5). The relative ordering of goals is consistent across

different MCMC chains; for example, the social-utility weight is highest for the social goal condition, lowest for the informational goal, and in the middle for the both-goal condition. In addition, the projected social-utility weight ϕ is inferred to be more on the informational-side (higher ϕ value) for the informational goal than for the social or both goal conditions. These patterns suggest that a lower-dimensional parameterization of the model may be available, though the posterior predictive fits and model comparison presented in the main text suggest that the model's parameterization has the appropriate flexibility necessary to account for our experimental data.

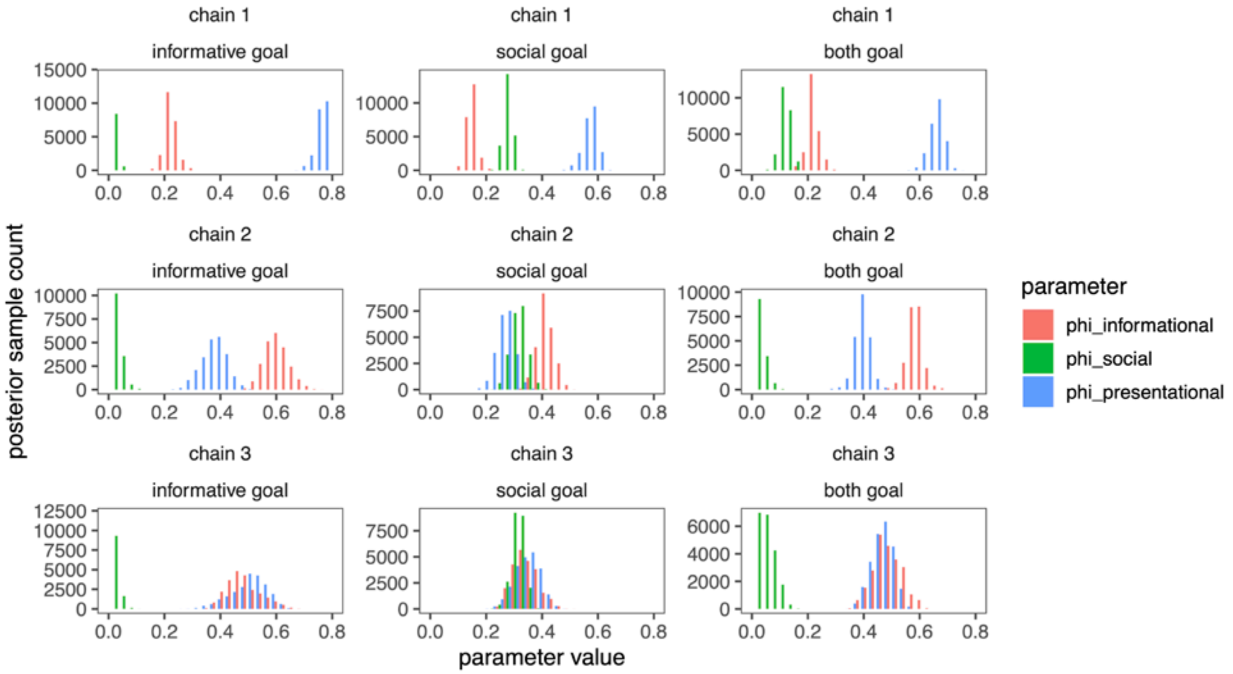

Figure 4 A goal-centric view of the utility-weight parameters. Columns denote different goal conditions and rows denote different MCMC chains. There are substantial inconsistencies in the posterior distributions over utility-weights. Social utility appears most consistent.

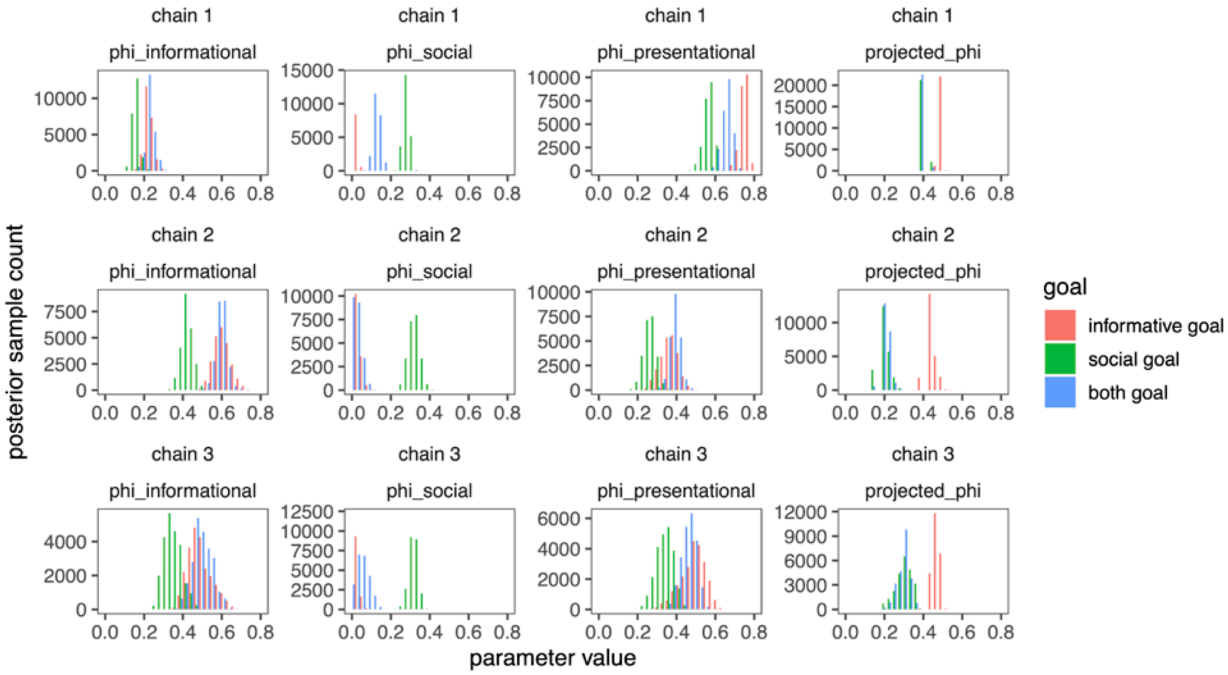

Figure 5 A utility-centric view of the utility-weight parameters. Relative orderings of the utility-weights across the different goal conditions are consistent.

Data Availability

Our model, preregistration of hypotheses, procedure, data, and analyses are available at .

Supplemental Figures

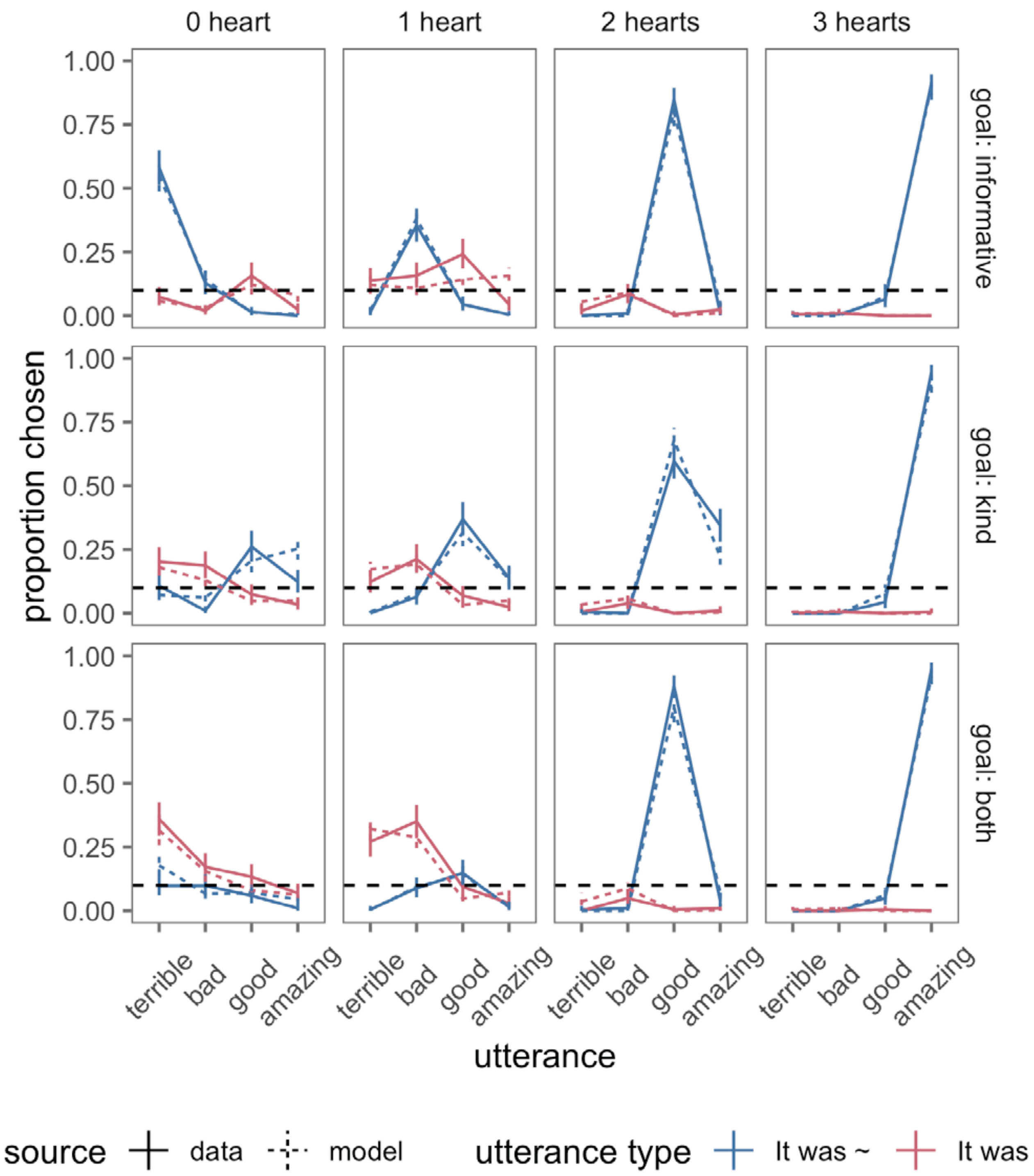

Figure 6 Experimental results (solid lines) and fitted predictions from the full model (dashed lines) for speaker production. Proportion of utterances chosen (utterance type – direct vs. indirect

– in different colors and words shown on x-axis) given the true states (columns) and speaker goals (rows). Error bars represent 95% confidence intervals for the data and 95% highest density intervals for the model. Black dotted line represents the chance level.

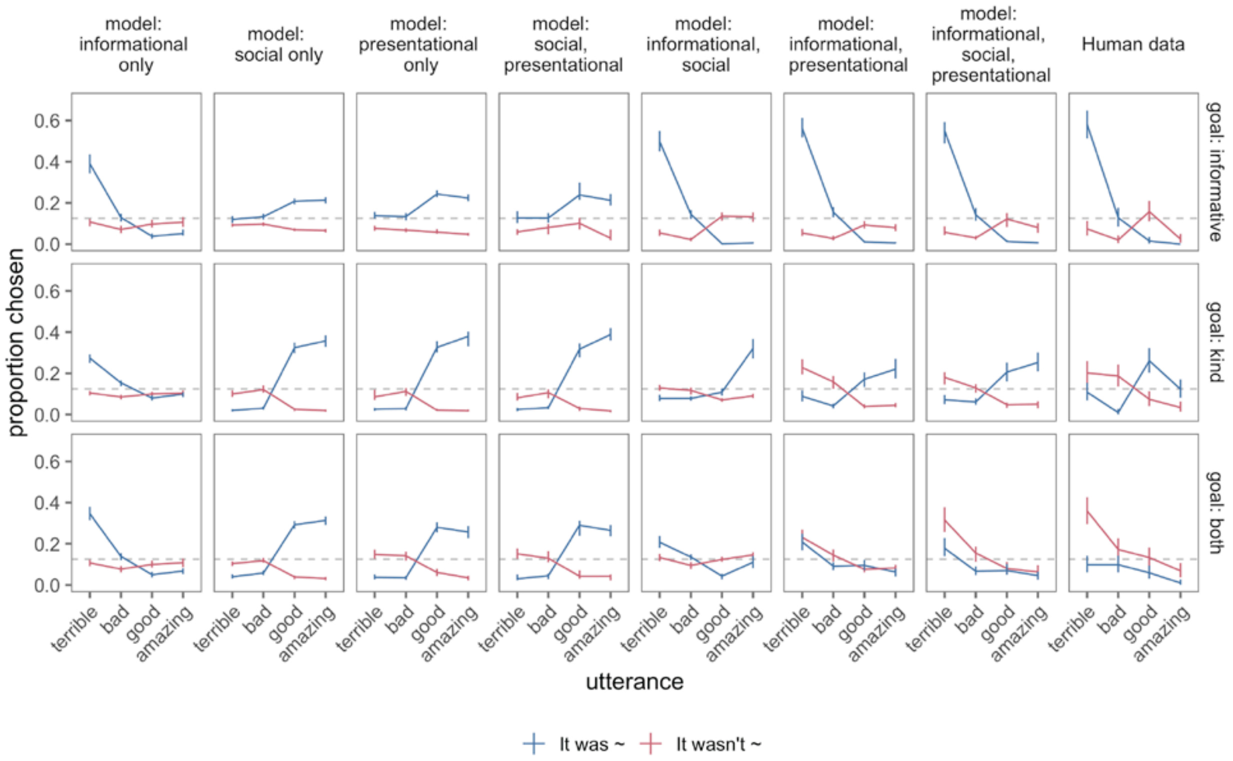

Figure 7 Comparison of predictions for proportion of utterances chosen by pragmatic speaker from possible model variants (left) and human data (rightmost) for average proportion of negation produced among all utterances, given true state of 0 heart and speaker with a goal to be informative (top), kind (middle), or both (bottom). Gray dotted line indicates chance level at 12.5%. Error bars represent 95% confidence intervals for the data (rightmost) and 95% highest density intervals for the models (left).

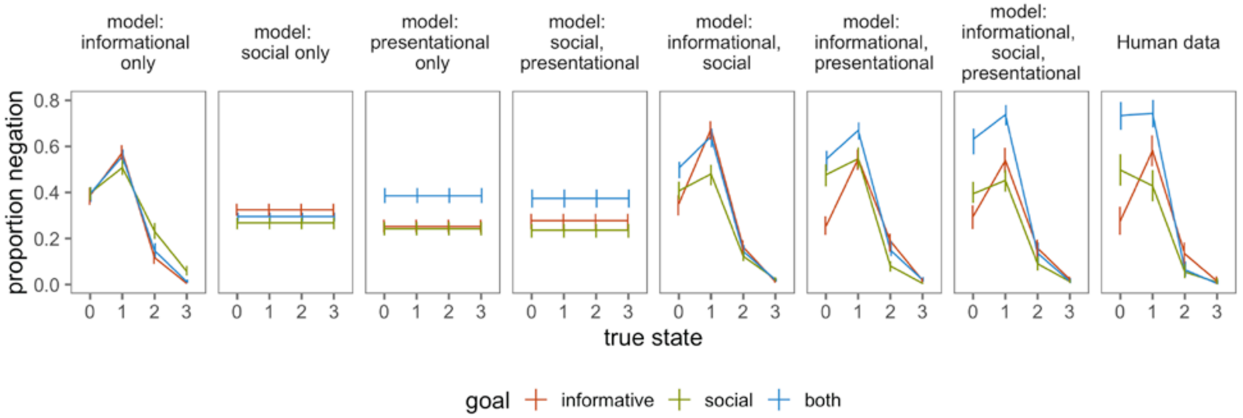

Figure 8 Experimental results (left) and fitted model predictions (right) for average proportion of negation produced among all utterances, given true states (x-axis) and goals (colors).
